# Lifestyle, Cardiovascular Drugs and Risk Factors in Younger and Elder Adults: The PEP Family Heart Study

**Published:** 2010

**Authors:** Peter Schwandt, Evelyn Liepold, Thomas Bertsch, Gerda-Maria Haas

**Affiliations:** 1 Arteriosklerose-Praeventions-Institut, Munich-Nuremberg, Germany; 2 Inst.Clin.Chem. Klinikum Nuremberg, Germany; 3 Ludwig-Maximilians-University of Munich, Munich. Germany

**Keywords:** Prevention, Cardiovascular disease, Ageing, Lifestyle habits, Germany

## Abstract

**Objectives:**

This study aimed to compare cardiovascular disease (CVD) risk factors, lifestyle habits and pharmacological treatment in two groups of elder adults with 20 years difference in their mean age.

**Methods:**

This study comprised 590 women including two groups with mean age of 42.4±5.5 vs. 66.5±4.0 years, and 486 men of two groups with mean age of 44.1±5.6 vs. 63.9±7.0 years. Data on physical examination, fasting blood analyses, 7-day dietary records, physical activity, smoking and actual medication use were recorded.

**Results:**

Compared with younger individuals, seniors had a more adverse risk factor profile in terms of abdominal obesity, overweight, hyperglycemia, hypertension, dyslipoproteinemia without differences in HDL-C. But this is not reflected by lifestyle behavior. Less than 2% of the elderly and 17% of the younger adults were current smoker. Furthermore, the pattern of physical activity was different in terms of more continuous sports in seniors contrasting with extremes between no sports and more than twice a week in the younger group. Seniors consumed significantly less carbohydrates including more monosaccharide and less polysaccharides, more alcohol and water. The intake of fat and protein was higher in elder women than in all other groups. One third of seniors took antihypertensive medications and 12% used lipid modifying drugs.

**Conclusions:**

Different levels of prevention against CVDs and their risk factors shall be considered for various age groups of population. The findings of this study emphasize on the necessity of preventive measures against smoking and physical inactivity in younger adults and dietary habits in seniors.

## INTRODUCTION

Physical inactivity, smoking and unhealthy nutrition have a major impact on the burden of coronary heart disease (CHD).^1^–^3^ Among women, adherence to lifestyle guidelines involving diet, exercise, and abstinence of smoking is shown to be associated with a very low risk of CHD.^4^ Recent meta-analyses demonstrated that better cardio-respiratory fitness is associated with lower risk of all-cause mortality as well as cardiovascular disease (CVD) including CHD. Moreover a causal link between dietary factors and CHD has been demonstrated.^5^,^6^ Therapeutic lifestyle change remains the preferred treatment of some risk factors as hypertriglyceridemia.^7^

Confirmation of the long-term results of major trials on lowering low density lipoprotein-cholesterol (LDL-C) is needed before widespread use of statins in apparently healthy individuals with average risk; actually healthy lifestyle including avoidance of smoking, prudent diet and regular exercise is a safe basis for prevention.^8^ However, this seems far from being established according to the recent postulation of “a lifeline for primary care”.^9^

Promise might come from the family-based CVD prevention program as EUROACTION which demonstrated that standards of preventive care can be improved in clinical practice resulting in healthier lifestyle changes and improve-ment of risk factors.^10^ To evaluate the actual need for lifestyle intervention in adult participants in the Prevention Education Program (PEP) Family Heart Study,^11^,^12^ we compared CVD risk profiles and lifestyle behavior of a sub-sample of 1,076 adults from two age groups.

## METHODS

After obtaining written informed consent, 1076 adults from two age groups were enrolled in this study. They consisted of 641 women, aged 42.4±5.5 and 66.5±4.0 years, as well as 435 men with 44.1±5.6 and 63.9±7.0 years of age. The 590 younger adults were selected from the parents participating in the 14 years’ prospective PEP Family Heart Study^11^ and the 486 elder adults were recruited by advertisement in Nuremberg. Exclusion criteria for PEP cohort were clinically apparent cardiovascular, metabolic, endocrine and malignant diseases while only seniors with a history of myocardial infarction and/or stroke were excluded. This procedure allows comparisons between presumably healthy younger individuals, i.e. parents of the first graders, who served as index persons for the PEP Family Heart Study, and elder subjects under care of their family physicians.

Over night fasted blood sampling was performed in central school buildings, stored and transported in cooled boxes to the laboratory. Weight, height, waist circumference (WC), blood pressure, total-, LDL-, and HDL- cholesterol, triglycerides (TG) and glucose were measured with standardized methods as described previously.^12^ Body mass index (BMI) was calculated as weight (Kg) divided by height squared (m^2^). Data on personal history, especially about cardiovascular, metabolic diseases and malignancies, taking lipid-modifying, antihypertensive and anti-diabetic medications, as well as lifestyle habits were recorded. For dietary habits, 7- day weighted dietary not questionnaires were completed and analyzed with PRODI 4 software.

The metabolic syndrome (MetS) was defined by using the criteria of the International Diabetes Federation (IDF)^13^ and Adult Treatment Panel III (ATPIII).^14^

### Statistical analysis

Continuous data are presented as means ± standard deviation (SD), and frequencies as percent.

Data were analyzed using the SPSS statistical package version 14.0 for windows (SPSS Inc., Chicago, USA). The significance level was set at p<0.05.

## RESULTS

In comparison with the younger group, fasting plasma glucose, total cholesterol, waist circumference and blood pressure were significantly higher in elder men and women (Table 1). Furthermore, elder women had higher TG, LDL-C and BMI values. HDL-C was not different between the 2 age groups but was higher among females than in males.

**Table 1:** Characteristics (mean±SD) of older and younger adults: the PEP Family Heart Study

	n	Younger men	n	Older men	n	Younger women	n	Older women
Age (years)	233	44.1±5.6	202	63.9±7.0***	357	42.4±5.5	284	66.6±4.0***
Glucose (mg/dL)	226	94.9±10.5	201	103.5±16.1***	350	90.8±10.1	284	99.4±16.2***
Cholesterol (mg/dL)	226	200.7±33.8	202	210.6±35.9***	350	186.6±33.2	283	231.9±39.3***
Triglycerides (mg/dL)	226	124.3±89.1	202	140.7±100.4	350	82.2±38.1	283	127.5±101.5***
HDL-Cholesterol (mg/dL)	226	50.1±10.6	202	51.6±11.4	350	63.9±15.2	283	63.5±14.3
LDL-Cholesterol (mg/dL)	223	126.2±29.0	198	132.5±30.1*	350	106.3±30.9	282	143.6±34.6***
Body mass index (kg/m^2^)	230	28.0±3.5	201	26.6±4.1	355	24.5±3.9	283	25.8±3.7***
Waist circumference(cm)	230	93,2±9.3	174	98.7±10.3***	357	84.2±10.2	279	93.4±11.1***
Systolic blood pressure (mmHg)	230	127.9±13.2	201	136.1±14.5***	357	115.8±11.8	283	134.5±19.7***
Diastolic blood pressure (mmHg)	230	85.6±0.1	201	83.03**	357	77.2±8.0	283	81.7±10.5***

Significance between men and women according to the age group:

* :p<0.05

** :p<0.01

*** :P<0.001

Table 2 demonstrates the prevalence of traditional CVD risk factors and of the metabolic syndrome (MetS). Prevalence of increased WC was considerably higher in elder than in younger adults. High blood pressure, elevated fasting plasma glucose and increased TG levels were 3-4 times more frequent in senior than in younger women. While the prevalence of low HDL-C (15-17%) was nearly identical among all groups, more than half of the seniors had increased LDL-C levels.

**Table 2:** Prevalence of cardiovascular risk factors and components of metabolic syndrome in younger and older adults:the PEP Family Heart Study

Men / Women	n	Younger men	n	Older men	n	Younger women	n	Older women
Mean age (years)		44.1±5.6		63.9±7.0		42.4±5.5		66.6±4.0
Waist circumference ≥94 cm / ≥ 80 cm a ≥102cm / ≥ 88 cm	230		174		357		263	
		38.7%		66.7%		58.5%		87.8%
		12.0%		26.7%		31.7%		63.4%
Body mass index > 30 kg/m^2^	230	11.3%	201	13.9%	357	7.8%	263	14.5%
High blood pressure ≥130/85 mm Hg a ≥140/90 mm Hg	230		201		357		283	
		52.6%		67.7%		15.1%		54.4%
		29.2%		29.2%		7.3%		26.4%
Fasting blood glucose ≥ 100 mg/dL a ≥ 110 mg/dL	226		201		350		284	
		31.4%		53.2%		14.0%		36.6%
		5.6%		20.8%		2.0%		14.8%
Total cholesterol >200 mg/dL	226		202		350		283	
		44.2%		58.4%		27.7%		75.7%
Triglycerides a >150 mg/dL	226		202		350		283	
		21.7%		21.7%		6.3%		25.4%
HDL-cholesterol a males <40 mg/dL/ females<50mg/dL	226		202		350		283	
		15.0%		14.9%		17.4%		16.6%
LDL- cholesterol > 130 mg/dL	223		198		350		282	
		41.3%		53.0%		20.9%		63.3%
Metabolic syndrome 3 of 5 components respectively WC plus any two components a	226		174		350		263	
		24.5%		36.6%		9.8%		36.3%
		7.3%		13.4%		4.5%		17.6%

a: indicates definition of the components of the metabolic syndrome according to IDF^13^ and ATPIII^14^ criteria

Nutritional data of the two age groups revealed that the daily consumption of energy was nearly identical though elder women consumed significantly more fat and protein than the younger women as well as men of both age groups (Table 3). The significantly lower consumption of carbohydrates in the elderly was mainly due to lower intake of polysaccharides while the younger adults have a higher consumption of fiber. Seniors had a significantly higher water consumption. Alcohol consumption was higher in the elderly than in the younger age group and in men than in women. There was a significantly higher energy intake (more than 6.5 % of energy) from alcohol in senior men.

**Table 3:** Daily intake of energy and nutrients in younger and older adults: the PEP Family Heart Study

	Men		Women	
	
	Younger n=233	Older n=202	Younger n=357	Older n=284
Kcal/day	2325 ± 487	2259 ± 445	1802 ± 382	1798 ± 380
Energy % fat	35.2 ± 5.0	34.8 ± 5.2	34.9 ± 5.2	36.3 ± 6.3**
SFA (g/day)	37.1 ± 11.3*	34.8 ± 11.0	29.2 ± 9.4	29.9 ± 10.0
MUFA (g/day)	32.9 ± 9.6	31.8 ± 10.0	24.5 ± 7.3*	25.9 ± 8.3
PUFA (g/day)	14.6 ± 5.1	14.6 ± 5.5	11.4 ± 4.9	11.9 ± 4.6
Energy % Carbohydrates	45.2 ± 5.8**	43.6 ± 6.9	48.0 ± 5.7***	45.0 ± 7.0
Monosaccharide (g/day)	38.6 ± 20.0	43.1 ± 23.0*	35.5 ± 16.3	39.1 ± 18.0
Disaccharides (g/day)	68.7 ± 30.1*	62.1 ± 28.7	62.9 ± 25.1**	57.2 ± 23.3
Polysaccharides (g/day)	142.2 ± 37.9***	125.0 ± 36.5	109.5 ± 27.9***	96.7 ± 26.6
Energy % Protein	15.0 ± 2.2	15.2 ± 2.6	14.8 ± 2.4	15.6 ± 2.7***
Energy % Alcohol	4.5 ± 4.7	6.5 ± 5***	2.4 ± 3.0	3.2 ± 3.9
Water (ml/day)	2741 ± 715	3052 ± 824***	2483 ± 784	2799 ± 786***

* p<0.05

** p<0.01

*** p<0.001

SFA: saturated fatty acid; MUFA: monounsaturated fatty acid; PUFA: polyunsaturated fatty acid

**Figure 1:**
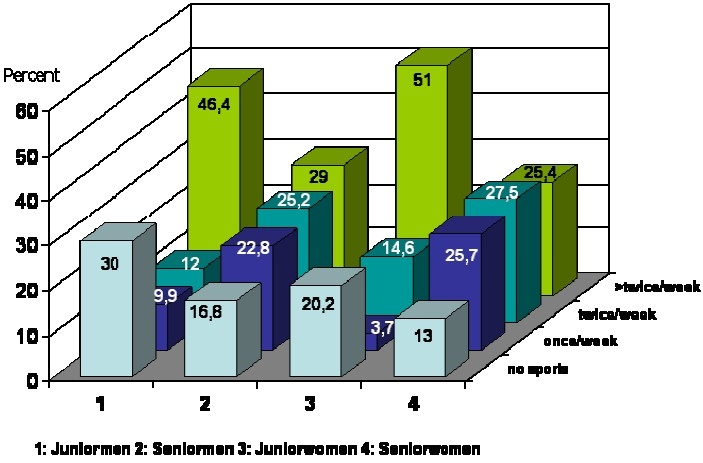
Frequency (%) of different levels of leisure time physical activity in younger (junior) and older (senior) adults : the PEP Family Heart Study

Figure 1 demonstrates that among elderly individuals, 17% of men and 13% of women had no sport activities, the corresponding figures were respectively 30% and 20% among the younger adults. About 50% of the younger age group reported to have regular exercise more than twice a week as compared to less than 30% of the elderly. Exercise of lower intensity was more regular in elderly than in younger individuals.

In the elder group, 1% of women and 2% of men reported to be current smoker; the corresponding figure among younger age group was respectively 13% and17%. Passive smoking was reported by 7% of men and 5% of women of the younger age group.

**Figure 2:**
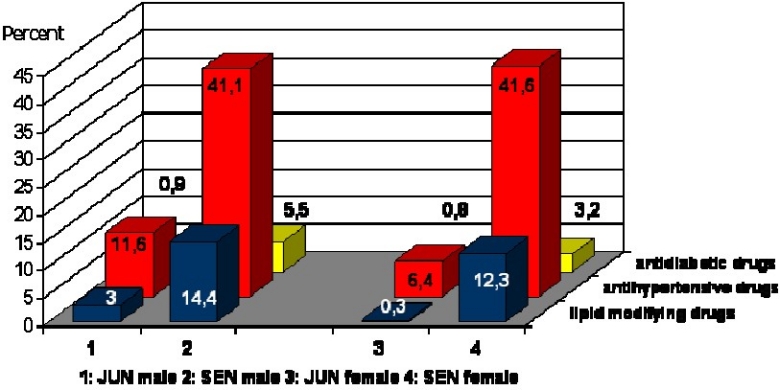
Frequency (%) of regular medication use in younger (junior) and older (senior) adults: the PEP Family Heart Study

As shown in Figure 2, more than 40% of the elderly reported taking anti-hypertensive medications, which is 4 to 6 times higher than the younger age group. In addition, lipid-modifying and anti-diabetic pharmacologic treatment was substantially higher in the elderly.

## DISCUSSION

This community-based study showed crosssectional differences between the two age groups of German adults in terms of lifestyle habits and cardiometabolic risk factors. Although the daily consumption of energy was nearly identical in both age groups, the dietary habits had significant differences. One of these differences was about the higher consumption of carbohydrates in terms of polysaccharides in younger than in the older age group. Fat and protein consumption was significantly higher in older women than in all other individuals. Seniors had a significantly higher alcohol and water consumption; the latter might reflect permanent advice to consume about 3 liters of energy- free drinks daily. In general, these findings emphasize on the necessity of paying more attention to improvement of dietary habits of elderly and in turn to secondary prevention of chronic diseases, notably CVDs.

Active and passive smoking was more frequent in younger than in older age groups. This reflects that more effort is needed to reduce tobacco use in younger age groups and to control environmental tobacco smoke.

Among the younger age group, nearly one third of men and one fifth of women reported that they had any leisure time physical activity, however nearly half of them reported that they were performing sports more than twice per week. Although, as expected, seniors had lower intensity of physical activity, but they had a more balanced profile of physical activity in terms of more than one session of physical activity per week. These differences might be due to the fact that the older age group studied is retired and/or handicapped by health problems while among the younger age group, the daily obligations only allowed between nothing and frequent sports. Rapid changes in lifestyle in an industrialized region with inevitable changes in transportation and workplace physical activity, as well as tendency to sedentary leisure time activities among newer generations might explain the low level of physical activity among the younger age group in this study.

Furthermore, in this study older individuals had a considerably worse cardiovascular risk profile than the younger adults in terms of LDL-hypercholesterolemia, abdominal obesity, metabolic syndrome, fasting glucose and hypertension (in women only). The higher prevalence of hypertension, fasting hyperglycemia and elevated waist circumference is due to more restrictive IDF^13^ definitions of the MetS components compared with the ATP III^14^ definitions. Using the IDF definition, the prevalence of abdominal obesity was nearly 30% higher in seniors than in juniors. Given that increased WC is an independent risk factor for CVD and a very good predictor of insulin resistance,^15^ seniors had an increased risk for consequences of insulin resistance. As about one fifth of the seniors had also increased TG, this combination designated as ‘hypertriglyceridemic waist’ (HTGW) might increase their risk additionally. HTGW was associated with increased risk of CVD after 7.5 years of follow-up in low-risk middle aged men^16^ and is even detectable from childhood.^17^ The TG-to- HDL-C ratio is an indicator of insulin resistance^18^ is slightly higher in seniors than in juniors.

## CONCLUSIONS

Different levels of prevention against CVDs and their risk factors shall be considered for various age groups of population. It should be more intensely communicated to the younger adults in order to demonstrate that getting older must not necessarily mean becoming more handicapped. Although the process of ageing increases the risk of chronic diseases, health promotion through lifestyle modification would be effective for various age groups. The findings of this study emphasize on the necessity of preventive measures against smoking and physical inactivity in younger adults and dietary habits in seniors.
